# Regulation of androgen receptor signaling by ubiquitination during folliculogenesis and its possible dysregulation in polycystic ovarian syndrome

**DOI:** 10.1038/s41598-017-09880-0

**Published:** 2017-08-31

**Authors:** Jung Jin Lim, Patricia D. A. Lima, Reza Salehi, Dong Ryul Lee, Benjamin K. Tsang

**Affiliations:** 1 Department of Obstetrics and Gynecology and Cellular and Molecular Medicine, University of Ottawa, Ottawa, Ontario, K1H 8L6 Canada; 2 State Key Laboratory of Quality Research in Chinese Medicine, Macau Institute for Applied Research in Medicine and Health, Macau University of Science and Technology, Avenida Wai Long, Taipa, Macao, China; 3 Chronic Disease Program, Ottawa Hospital Research Institute, Ottawa, Ontario, K1H 8L6 Canada; 4 Fertility Center of CHA Gangnam Medical Center, College of Medicine, CHA University, Seoul, 135-913 Korea; 5 Department of Biomedical Science, College of Life Science, CHA University, Seoul, 135-081 Korea; 6 Department of Biomedical Science, Graduate School of Biomedical Science and Engineering, Hanyang University, Seoul, 133-791 Korea

## Abstract

Although chronic hyperandrogenism suppresses antral follicular development, a phenomenon often observed in polycystic ovarian syndrome (PCOS), whether and how deregulation of androgen receptor (AR) signaling is involved, is not well understood. In the present study, we examined the role of ring finger protein 6 (RNF6) in AR ubiquitination and the possible dysregulation in the expression and actions of growth differentiation factor 9 (GDF9) and kit-ligand (Kitlg) in a chronic androgenized PCOS rat model. 5α-dihydrotestosterone (DHT) treatment *in vivo* inhibited antral follicle growth, a response mediated through increased RNF6 content, suppressed K63- but increased K48-linked AR ubiquitination as well as the mRNA expression and content of soluble KIT-L (sKitlg) and content of GDF9. These androgenic responses were attenuated by gonadotropin treatment *in vivo*. Growth of antral follicles from DHT-treated rats *in vitro* was significantly slower when compared to those of control but was significantly enhanced by exogenous GDF9, suggesting the DHT-induced antral follicular growth arrest is in part the results of GDF9 suppression. Our findings indicate how hyperandrogenism modulates RNF6 content and subsequently AR ubiquitination, resulting in antral follicle growth arrest in a chronically androgenized PCOS rat model.

## Introduction

Androgens stimulate granulosa cell proliferation and promote preantral follicle growth in the mammalian ovary^1–4^, but suppress later stages of follicular development through induction of granulosa cell apoptosis, symptoms often associated with ovarian dysregulation evident in hyperandrogenic anovulation^5, 6^. Polycystic ovarian syndrome (PCOS) is a heterogeneous syndrome affecting 10% of women in reproductive age and accounts for 75% of anovulatory fertility. It is associated with antral follicle growth arrest, suppressed proliferation and enhanced apoptosis of granulosa cells and hyperandrogenemia^7^. The molecular and cellular mechanisms involved in antral follicular growth arrest in PCOS are not well understood.

Androgen receptor (AR) plays important regulatory roles in ovarian follicular development. It is well established that the cellular actions of androgens are mediated via its binding to and activation of AR, which regulates gene transcription in androgen-dependent cell growth and proliferation. AR signaling is regulated by a variety of posttranslational modifications, including ubiquitination, phosphorylation, acetylation, methylation and sumoylation^8^. Protein ubiquitination involves the binding of ubiquitin to substrates as single ubiquitin (mono-ubiquitination) or as ubiquitin chain (poly-ubiquitination). The ubiquitination process involves sequential action of ubiquitin-E1, E2 and E3, whereas E3 is a determinant of substrate specificity^9, 10^. Ubiquitin contains seven lysine residues K-6, 11, 27, 29, 33, 48, and 63 through which the ubiquitination chain extends^11^. It is well established that polyubiquitination at K48 and K63 leads to protein degradation by 26 S proteasome^12^ and transcriptional activation^13^, respectively. Small nuclear RING (Really Interesting New Gene) finger proteins are E3 ligases and are nuclear receptor co-regulators^14, 15^. Ring finger protein 6 (RNF6), a member of this family, induces AR ubiquitination^8^. RNF6 is believed to promote AR transcriptional activity^16^ or AR proteasome degradation^17^ and it appears to be cell type-specific and dependent on stage of cellular differentiation. We have recently demonstrated the role of RNF6 in AR signaling in the regulation of granulosa cell fate during preantral follicle growth. RNF6 is a positive mediator in androgen-induced AR polyubiquitination, *Kitlg* mRNA expression and granulosa cell proliferation *in vitro* ^18^. However, whether and how RNF6 is important in the dysregulation of follicular growth in PCOS is not known.

Follicular cell proliferation and survival are tightly regulated by follicular factors including granulosa cell-derived factor Kitlg^19, 20^ and the oocyte-derived factor growth differentiation factor 9 (GDF9)^21^. Depending on the stage of follicular development, androgens could promote or suppress ovarian follicle growth^22, 23^. While androgens enhance preantral follicle growth by stimulating the GDF9^24, 25^, chronic androgenic stimulation induces antral follicle growth arrest and polycystic ovarian syndrome phenotypes in the rats by GDF9 down-regulation^26^. Kitlg signals through its receptor Kit in the oocyte and stimulates oocyte growth and folliculogenesis. Androgen is known to up-regulate granulosa cell expression of Kitlg and its action in the ovarian follicle^1^. Although GDF9 and Kitlg are known to mediate androgen action on follicular development, the cellular mechanisms involved in the AR ubiquitination induced by androgen are not completely understood.

The overall objective of the present studies was to examine the androgenic regulation of RNF6 expression and the role of RNF6-mediated AR ubiquitination in the dysregulation of ovarian follicular development, using a chronically androgenized rat PCOS model. In addition, whether and how these processes alter the expression of GDF9 and Kitlg was determined. We hypothesized that androgen-induced ovarian follicular growth is dependent on site-specific RNF6-mediated AR polyubiquitination, which determines AR transcriptional activity (K63; *Kitlg* mRNA expression) and AR stability and abundance (K48). During chronic androgenization, increased RNF6-mediated AR ubiquitination (K48) results in enhanced AR degradation and decreased Kitlg expression, GDF9 down-regulation and antral follicular growth arrest. Our studies suggest that antral follicle growth arrest observed in PCOS may in part be associated with androgen-induced, RNF6-mediated AR (K48) polyubiquitination and degradation and loss of granulosa cell proliferative response.

## Materials and Methods

### Reagents

Leibowitz L-15, α-MEM, streptomycin and penicillin, fetal bovine serum and trypsin were purchased from Life Technologies (Carlsbad, CA, USA). Bovine insulin, human transferrin, ascorbic acid, sodium selenite anhydrous, L-glutamine, sodium pyruvate, agarose (low gelling temperature), HEPES, equine chorionic gonadotropin (eCG) were purchased from Sigma (St Louis, MO, USA) and 5α-dihydrotestosterone (DHT) was purchased from Steraloids (Newport, RI, USA). Primary and secondary antibodies used in the present studies are shown in Supplementary Table 1. Protein A/G PLUS-agarose (sc-2003) was purchased from Santa Cruz Biotechnology (Santa Cruz, CA). Reagents for SDS-PAGE were supplied by Bio-Rad Laboratories (Mississauga, ON, Canada). RNeasy mini-kit and Quanti-Tect SYBR Green PCR kit were purchased from QIAGEN (Mississauga, ON, Canada). Primers used in the present studies are summarized in Supplementary Table 2. Random decamer primers were purchased from Ambion (Austin, TX, USA). Ribonuclease inhibitor and deoxynucleotide triphosphate were purchased from Fermentas (Burlington, ON, Canada). Moloney murine leukemia virus reverse transcriptase was purchased from Promega (Madison, WI, USA). All set of PCR primers were purchased from Integrated DNA Technologies (Redwood, CA, USA).

### DHT-treated rat PCOS model

Female Sprague Dawley rats were obtained from Charles River Canada (Montréal, Québec, Canada) and maintained under standard conditions. All animal procedures were carried out in accordance with the guidelines of the Canadian Council on Animal Care and approved by the University of Ottawa Animal Care Committee. Briefly, 21 days-old rats were divided into two groups [control (10 per replicate), DHT (15 per replicate); n = 3 replicates] and implanted subcutaneous for 30 days with SILASTIC brand capsules (Dow Corning Corp., Midland, MI, USA) containing 7.5 mg DHT (continuous daily release: 83 µg). Control rats received identical capsules without DHT. Granulosa cells were isolated from “preantral follicles” (also include early stage antral follicles; 110–150 µm) and “antral follicles” (150–200 µm) for protein and mRNA analyses.

### Follicle isolation and culture

Antral follicles (diameter, 150–200 µm) from CTL or DHT implanted rats were isolated in Leibowitz L-15 medium with BSA (0.1%, wt/vol), using 28.5-gauge needles (Becton Dickinson and Co., Franklin Lakes, New Jersey) under a stereomicroscope. Follicles with intact basement membranes and theca layers were individually cultured as described previously^27^. Depending on the design of the experiments, hormonal treatments were initiated at the beginning of culture. Follicular diameter was measured daily as the average distance between the outer edges of the basement membrane in 2 perpendicular planes and results were expressed as the change in follicular volume. Follicular volume was calculated according to the formula for the volume of a sphere: volume = 4πr^3^/3, where r is radius. The percent change in follicular volume on day *n* of culture is defined as the volume difference between day *n* and day 0 (the day of follicle isolation) expressed as a percentage of the volume at day 0. The culture medium was changed every other day.

### Western blot analysis

Procedures for protein extraction and Western blotting have been described previously^28^. Briefly, collected follicles were lysed and protein content was determined. Equal quantities of protein lysate (40 µg) were subjected to SDS-PAGE and transferred onto a nitrocellulose membrane. The membrane was incubated (1 h, RT) in Tris-buffered saline 0.1% tween 20 (TBST) and non-fat dry milk (5%). The membranes were then incubated (4 °C, overnight) with Rabbit anti-rat AR antibody (1:5000 dilution), GAPDH antibody (1:8000 dilution), rabbit anti-rat RNF6 antibody (1:5000 dilution), mouse anti-rat Kitlg antibody (1:5000 dilution) and then with Fluor-680 labeled secondary antibodies (1:10000; 1 h, room temperature). After washing three times with TBST, immunoreactive bands were visualized with the Odyssey infrared imaging system (LI-COR, Lincoln, NE) at a wavelength of 700 nm. The protein signals were densitometrically scanned and quantified, using AlphaEaseFC (Alpha Innotech, CA) and normalized with GAPDH expression.

### Immunoprecipitation

Cellular fractionation was performed as described previously^29^. Isolated follicles (~300) were harvested and lysed in a buffer (0.25 M sucrose, 10 mM Tris pH 7.5, 1 mM EDTA). In immunoprecipitation (IP) studies, cell lysates were pre-cleared (1 h) with protein A/G PLUS-agarose and supernatants were incubated with AR antibody (4 °C, overnight) and treated with protein A/G PLUS-agarose (4 °C, 4 h). Immunoprecipitates were washed three times with 1 mL TBS. After heating (95 °C, 5 min), co-immunoprecipitates (K48 or K63 ubiquitin) were detected by Western blot.

### Real-time quantitative PCR analysis

Granulosa cells were released from preantral and antral follicles by follicular puncture with a 26.5-gauge needle^28^ and total RNA was extracted with the RNeasy mini kit, according to the manufacturer’s instructions. Extracted RNA (200 ng) was reverse transcribed into cDNA with the Sensiscript RT kit. Real-time quantitative PCR analysis was performed as previously described^30^. The primers which were designed to detect sKitlg (NM_021843.4), but not mKitlg used for SYBR Green real-time quantitative PCR analysis are shown in the Supplementary Table 2. The thermal cycling conditions comprised an initial denaturation step at 95 °C for 10 min and 40 cycles at 95 °C for 30 sec, 52 °C (sKitlg) or 56 °C (GAPDH) for 30 sec, and 72 °C for 30 sec. Data were analyzed by the 2^-∆∆CT^ method, presented as the fold change in mRNA abundance normalized against the GAPDH gene and expressed relative to the respective control.

### Immunofluorescence (IF) microscopy

IF were performed as described previously with modifications^31^. After 4% paraformaldehyde (PFA) fixation, ovarian tissues embedded in paraffin and cut into 5 µm thick sections were washed three times in PBS. Slides were blocked with 3% BSA and incubated (overnight, 4 °C) with primary antibodies against rabbit anti-rat AR monoclonal antibody (1:100 dilution) or rabbit anti-rat RNF6 polyclonal antibody (1:100 dilution) or mouse anti-rat Kit ligand monoclonal antibody (1:100 dilution) or goat anti-human GDF9 polyclonal antibody (1:100 dilution). After washing three times with PBS, slides were incubated (1.5 h, room temperature) with Alexa Fluor 488 or 594 fluorescence-conjugated secondary antibodies (1:200 dilution) and mounted on slides with ProLong gold anti-fade reagent with DAPI) and optical images were obtained using a fluorescence microscope (Olympus, Melville, New York).

### Statistical analysis

All data were analyzed using GraphPad Prism 5.0 statistical software (San Diego, CA). Results are expressed as mean ± SEM of at least three independent experiments as detailed in the figures. One-way or two- ANOVA were used to assess the effects and interactions of one and two variables and multiple comparisons were achieved by a Bonferroni *post-hoc* test. Significant difference was inferred at p < 0.05.

## Results

### Chronic DHT treatment inhibits rat antral follicle growth *in vivo*

**Figure 1:**
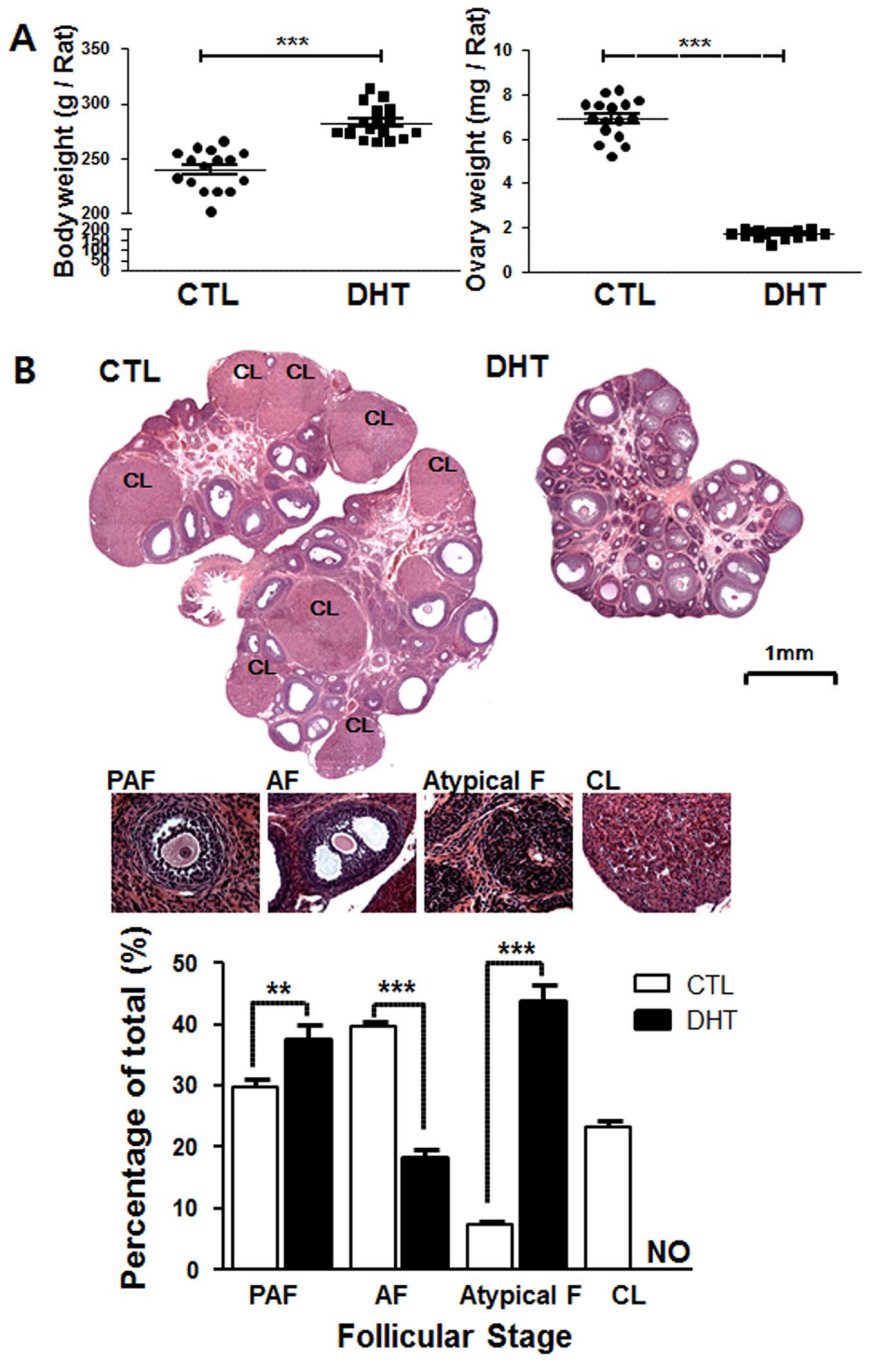
DHT inhibits antral follicle growth and induces ovarian and body weight changes in a PCOS rat model. (**A**) 21 days old female rats were randomly divided into two experimental groups (CTL and DHT) and subcutaneously implanted for 30 days with a capsule with continuous DHT release. Sham animals received an implant without DHT. Results are expressed as mean ± SEM of 15 rats (n = 3 replicates each with 5 rats per group). Data were analyzed by Student “t” test. Body weight: ***P < 0.001 (vs. CTL). Ovarian weight: ***P < 0.001 (vs. CTL). **(B)** Hematoxylin and eosin staining of CTL and DHT-treated ovaries. DHT-treated rat ovaries showed smaller ovarian size and contain lower numbers of antral follicles. Results are expressed as mean ± SEM of 15 ovary (n = 3 replicates each with 5 per group). Data were analyzed by two-way ANOVA and Bonferroni post hoc test. Preantral and early antral follicle (PAF) group: **P < 0.001 (vs. CTL). Antral follicle (AF) group: ***P < 0.001 (vs. CTL). Atypical follicle (Atypical F) group: ***P < 0.001 (vs. CTL). CL, corpus luteum; NO, not observed.

Using a rat model with DHT implant for 30 days, we first investigated the influence of chronic androgen treatment on body weight, ovarian weight and morphology, and follicular dynamics *in vivo* (Fig. 1). Although DHT-treated rats had a significantly higher body weight than those with capsules without DHT (CTL; *P* < 0.001), a marked reduction in overall ovarian weight and size was observed in the DHT-group (*P* < 0.001; Fig. 1A). The ovaries of control rats generally contained ovarian follicles in different stages of development in a single visual field. Theca and granulosa cell layers had normal appearance as well as the presence of corpus lutea. Ovaries of the DHT-treated rats contained a significantly higher number of preantral or early antral follicles (PAF; *P* < 0.05). There were significantly less large antral follicles (AF; *P* < 0.01) and no corpus lutea. In addition, the number of condensed atypical follicles containing primarily granulosa cells^26^ was significantly increased in the DHT-treated rats (*P* < 0.001 vs. CTL; Fig. 1B). Taken together, these findings suggest that chronic androgen is a negative regulator of antral follicular growth and ovulation *in vivo*.

### RNF6 content is increased while androgen receptor content is down-regulated in antral follicles of DHT-treated rats

**Figure 2:**
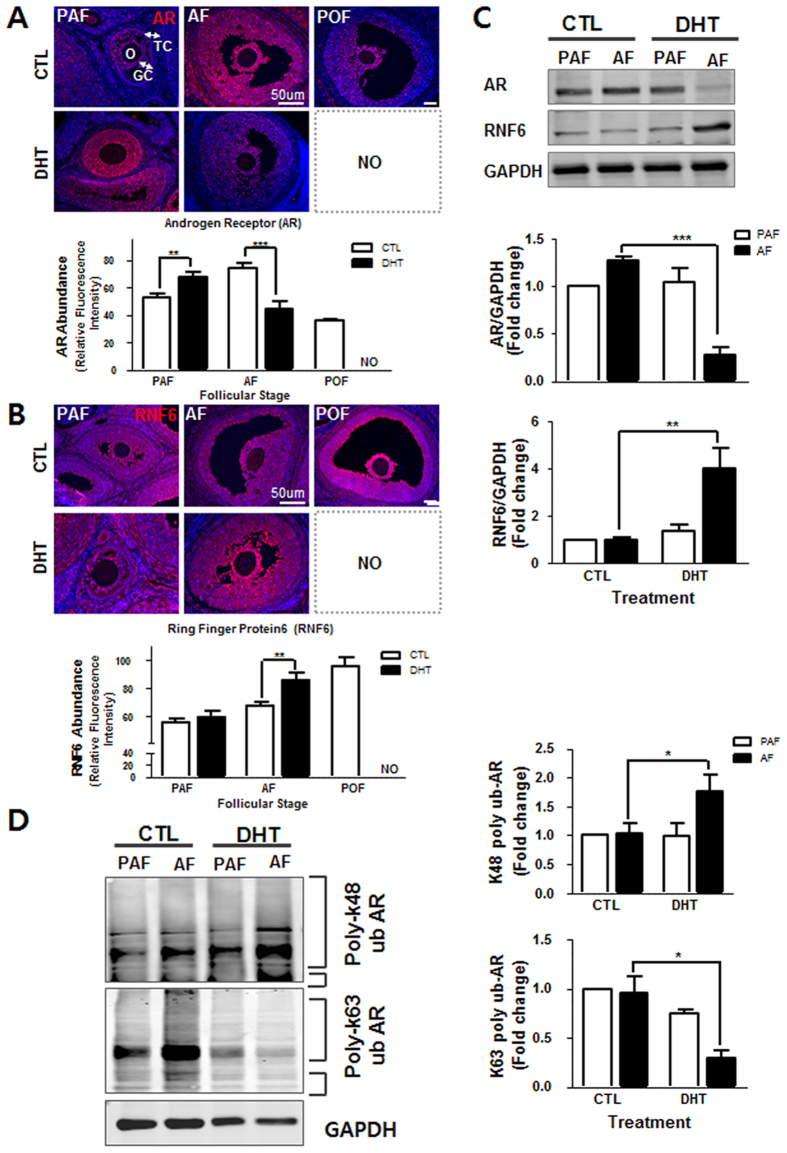
Chronic androgenization increases RNF6 levels and K48-linked AR poly-ubiquitination, but decreases AR content and suppresses antral follicle growth *in vivo*. **(A)** Ovarian tissue sections from CTL and DHT-treated rats were immunostained with anti- AR (red; IF), analyzed and quantified using the ImageJ software. Results are expressed as mean ± SEM (n = 3). Data were analyzed by 2-way ANOVA and Bonferroni post-hoc test. PAF group: **P < 0.01 (vs. CTL). AF group: [***P < 0.001 (vs. CTL). POF, pre-ovulatory follicle; O, oocyte; GC, granulosa cells; TC, theca cells **(B)** As in AR experiment (A), RNF6 content was analyzed by IF. Results are expressed as mean ± SEM (n = 3). Data were analyzed by 2-way ANOVA and Bonferroni post-hoc test. AF group: **P < 0.01 (vs. CTL). **(C)** Pre-antral or antral follicles isolated from CTL and DHT-treated rats were lysed and AR and RNF6 contents were assessed by Western blot. Results are expressed as mean ± SEM (n = 3). Data were analyzed by 2-way ANOVA and Bonferroni post-hoc test. In RNF6 analysis, PAF group: *P < 0.05 (vs. CTL). AF group: ***P < 0.001 (vs. CTL). In AR analysis, AF group: ***P < 0.01 (vs. CTL). **(D)** Preantral or antral follicles isolated from CTL and DHT-treated rats were lysed and immunoprecipitated using anti-AR, and the precipitate was immunoblotted with anti-K63 and anti-K48 ubiquitin antibody. Results are expressed as mean ± SEM (n = 3) and analyzed by 2-way ANOVA and Bonferroni post-hoc test. *P < 0.01 (vs. CTL).

We next compared the relative expression of AR and RNF6 at different stages of follicular development by immunofluorescence (IF) and Western blot (WB). Both AR and RNF6 were primarily localized in the granulosa cell layer (Fig 2A and B). The fluorescence intensity of AR at the antral follicular stage in DHT-treated rats was significantly lower than that in the controls (*P* < 0.001). Similarly, AR contents in the antral follicles decreased markedly following DHT treatment (*P* < 0.01 vs. CTL; Fig. 2C). However, in contrast to AR levels, RNF6 levels were increased in DHT-treated rat at the antral follicular stage, (*P* < 0.01 vs. CTL; Fig. 2C). Our findings indicate that AR and RNF6 are differentially expressed in antral granulosa cell layer during androgen-induced antral follicular growth arrest.

### DHT-induced antral growth arrest is associated with K63-linked poly-ubiquitination of AR and increased K48-linked poly-ubiquitination of AR

Previous studies on the prostate gland suggest that RNF6 directly modulates AR transcriptional activity and AR proteasome degradation during androgen-induced cell proliferation and apoptosis, respectively^16^. Recently, we have demonstrated that DHT increases AR polyubiquitination in granulosa cells from immature follicles at lysine residue 63 but not at lysine residue 48^18^. Mono-ubiquitination of AR at K63 and K48, often associated with intracellular transport, was not significantly affected by DHT. Androgen markedly increased K63-linked AR poly-ubiquitination in granulosa cells and this response was significantly inhibited by RNF6 silencing *in vitro* ^18^. To determine whether chronic androgen treatment *in vivo* influence follicular development through its RNF6-mediated AR actions, we compared K63- and K48- AR poly-ubiquitination by co-precipitation at pre-antral and late antral follicular stage without/with DHT treatment. AR in antral follicles from DHT-treated rats exhibited a marked decrease in K63-linked poly-ubiquitination (*P* < 0.05 vs. CTL; Fig. 2D), while the opposite was true in K48-linked polyubiquitination (*P* < 0.05 vs. CTL; Fig. 2D). The above observations suggest that androgen plays an important role on K48-linked AR poly-ubiquitination in the regulation of AR stability in the antral follicles and the highly expressed RNF6 in antral follicles might be positively involved in regulation of AR degradation.

### Gonadotropin down-regulates RNF6 content, differentially modulates AR K63- and K48-linked poly-ubiquitination and restores AR content in antral follicles of DHT-treated rats

**Figure 3:**
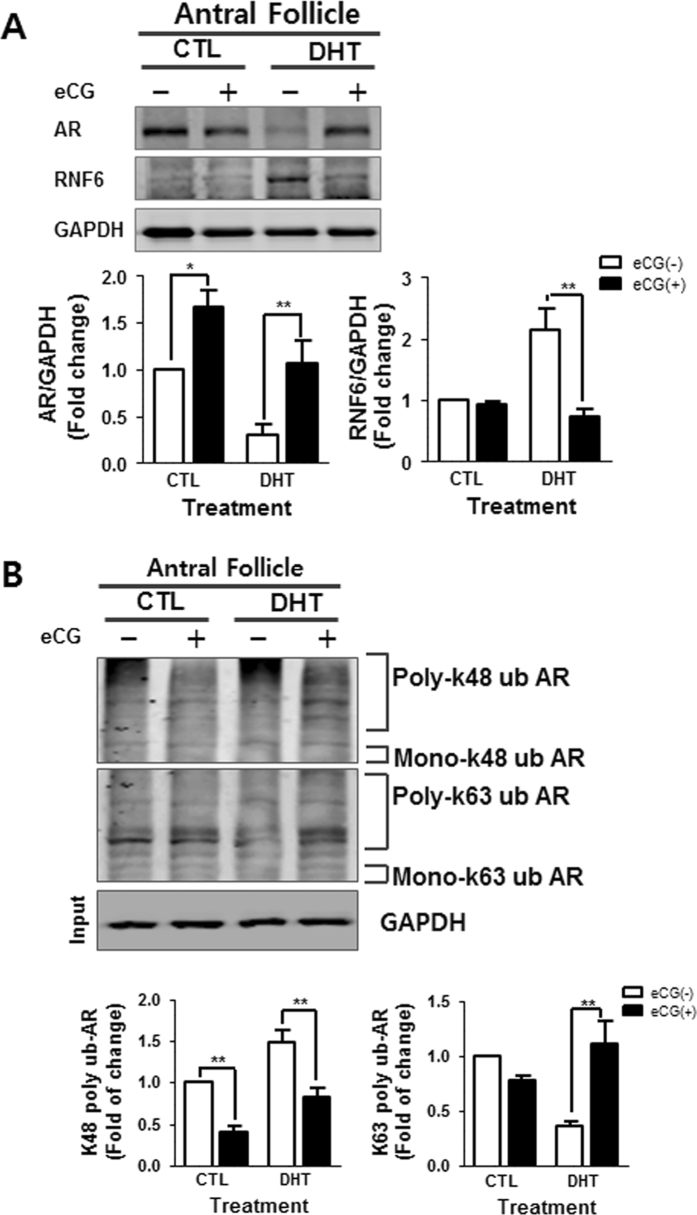
Gonadotropin up-regulates AR content and K63-AR ubiquitination, but down-regulates K48- AR ubiquitination and RNF6 content in the PCOS rat model. (**A**) After eCG (10IU, i.p.) injection, antral follicles isolated 48 h thereafter from CTL and DHT-treated rats were lysed and AR and RNF6 content were assessed by Western blot. Results are expressed as mean ± SEM (n = 3). Data were analyzed by 2-way ANOVA and Bonferroni post-hoc test. CTL group: [AR: *P < 0.05 (vs. eCG)]. DHT group: [AR: **P < 0.01 (vs. eCG); RNF6: **P < 0.01 (vs. eCG)]. (**B**) After eCG injection (10 IU, i.p.), antral follicles isolated from CTL and DHT-treated rats 48 h thereafter were lysed and immunoprecipitated using anti-AR, and the precipitate was immunoblotted with anti-K63 and anti-K48 ubiquitin antibody. Results are expressed as mean ± SEM (n = 3) and analyzed by 2-way ANOVA and Bonferroni post-hoc test. In CTL group: [K48-linked AR poly-ubiquitination **P < 0.01 (vs. eCG + )]. In the DHT group: [K48-linked AR poly-ubiquitination **P < 0.01 (vs. eCG + ); K63-linked AR poly-ubiquitination **P < 0.01 (vs. eCG + )].

Controlled gonadotropin stimulation has been reported to be beneficial in the management of anovulatory infertility associated with polycystic ovary syndrome in subjects non-responsive to clomiphene^32^. Our previous studies have shown that deregulation of ovarian follicular growth in a DHT-induced PCOS rat model could be restored by gonadotropin administration^33, 34^. To determine the influence of gonadotropin on androgen-induced, RNF6-mediated AR poly-ubiquitination, eCG was injected into DHT-treated rats and its controls, and changes in AR and RNF6 contents and poly-ubiquitination of AR at K48 and K63 were determined. eCG significantly decreased RNF6 levels (*P* < 0.01 vs. CTL; Fig. 3A) and restored the AR contents (*P* < 0.01 vs. CTL; Fig. 3A) in antral follicles from DHT-treated group *in vivo*. In addition, the gonadotropin significantly increased AR poly-ubiquitination at the K63 site but decreased that at K48 site in the antral follicles compared to DHT-treated rat without eCG treatment (*P* < 0.01 vs. CTL; Fig. 3B). Our findings suggest that gonadotropin is an important regulator of RNF6 and AR expression in the antral follicles and that it modulates androgen-induced antral follicle growth arrest possibly by restoring poly-ubiquitination of AR at K63 but suppressing that at K48.

### Gonadotropin enhances sKitlg expression and restores antral follicle growth in DHT-treated rats

**Figure 4:**
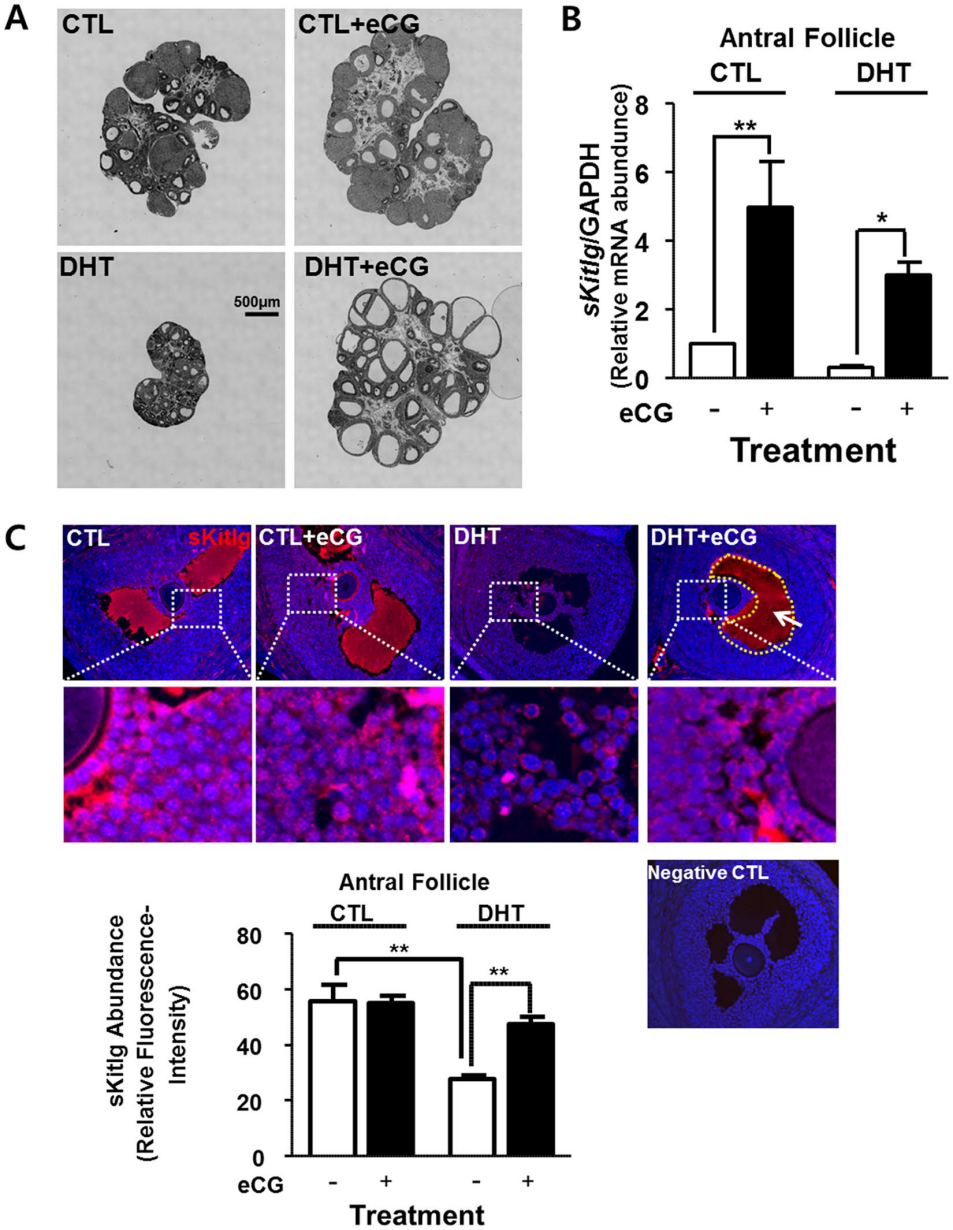
Gonadotropin enhances *sKitlg* expression in the antral follicles and restores folliculogenesis in DHT-treated rats. **(A**) Forty eight hours after eCG injection (10 IU, i.p.), ovaries from CTL and DHT-treated rats were sectioned and stained with hematoxylin and eosin. eCG increased of ovarian size and antral follicle number in the DHT -treated rats. **(B)** After eCG injection (10 IU, 48 h), antral follicles isolated from CTL and DHT-treated rats were lysed and soluble-kit ligand mRNA abundances were assessed by quantitative RT-PCR. Results are expressed as mean ± SEM (n = 3). Data were analyzed by 2-way ANOVA and Bonferroni post-hoc test. CTL group: **P < 0.01 (vs. eCG); DHT group: **P < 0.01 (vs. eCG). **(C)** After eCG injection, ovarian tissue sections from CTL and DHT-treated rats were immunostained for soluble-kit ligand, analyzed and quantified using the ImageJ software. Results are expressed as mean ± SEM of 15 follicles from 3 experimental replicates. Data were analyzed by 2-way ANOVA and Bonferroni post-hoc test. DHT group: **P < 0.01 (vs. eCG); Without eCG group: **P < 0.01 (CLT vs. DHT).

As shown in Fig. 4A, eCG restores folliculogenesis in the DHT-treat rats which otherwise exhibit antral follicles arrest. Treatment with eCG increased number of antral follicles and ovarian size, which was similar to non-treated control group. Moreover, eCG restored *sKitlg* mRNA abundance (*P* < 0.05 vs. CTL; Fig. 4B) and protein contents (*P* < 0.01 vs. CTL; Fig. 4C) in the antral follicles from DHT-treated group. These findings suggest that gonadotropin up-regulates antral follicle growth in chronically androgenized rats, possibly by modulating the AR signaling via site-specific AR poly-ubiquitination and enhancing granulosa cell soluble-kit ligand expression.

### DHT suppresses GDF9 expression and antral follicle growth *in vivo*

**Figure 5:**
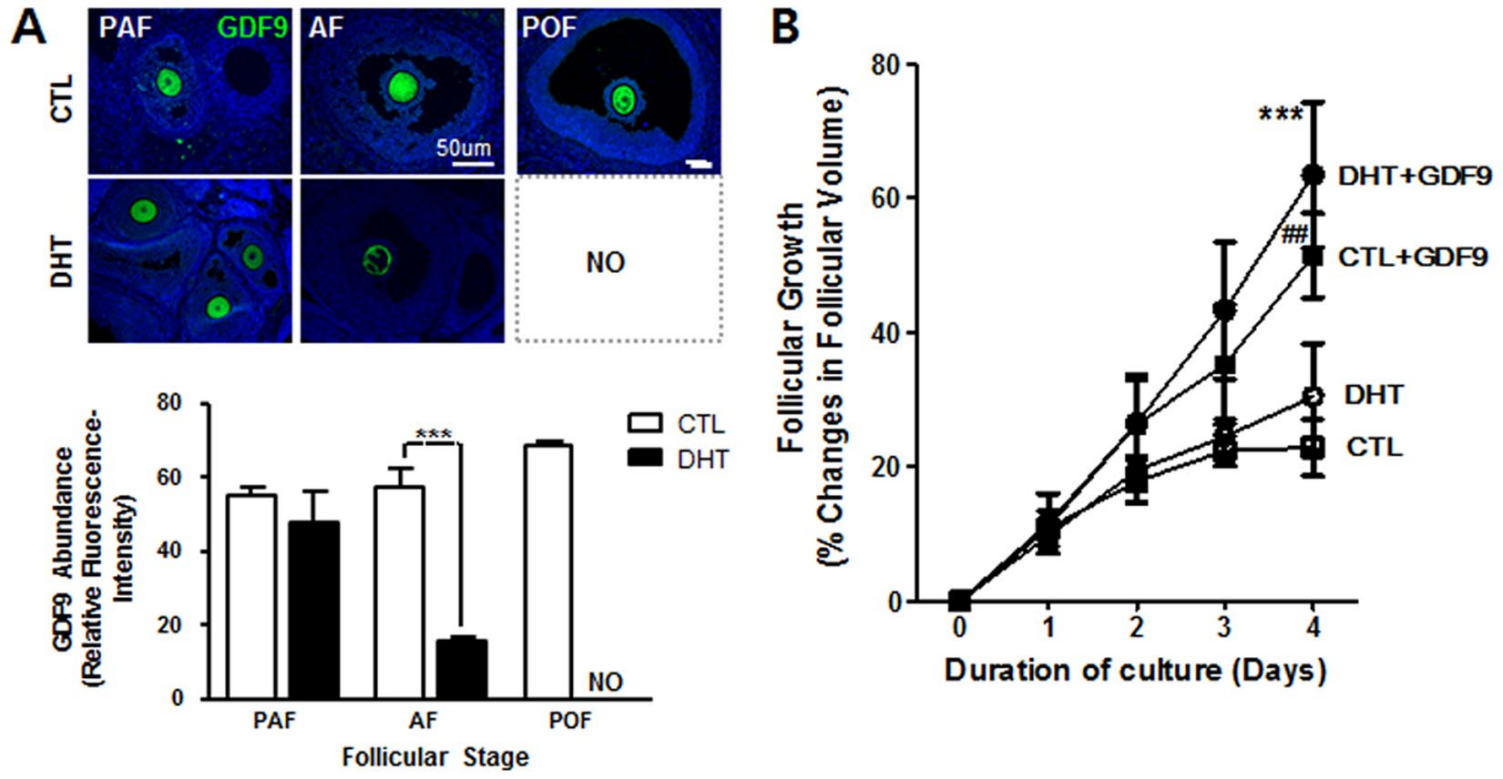
DHT suppresses GDF9 expression and the antral follicle growth *in vivo*, responses attenuated by exogenous GDF9 *in vitro*. **(A)** Ovarian tissue sections from CTL and DHT-treated rats were immunostained for GDF9 analyzed and quantified using the ImageJ software. Results are expressed as mean ± SEM of 15 follicles from 3 experimental replicates. Data were analyzed by 2-way ANOVA and Bonferroni post-hoc test. AF group: ***P < 0.001 (vs. CTL) **(B)** Antral follicles (150–200 um) isolated from CTL and DHT-treated rats were cultured for up to 4 days in the absence and presence of GDF9 (100 ng /ml). Changes in follicular volume (assessed daily) were expressed as mean ± SEM of 30 follicles from 3 experimental replicates. [***P < 0.001 (DHT + GDF9 vs. DHT); ^##^P < 0.01 (CTL + GDF9 vs. CTL)].

GDF9 is expressed in the mammalian oocyte throughout folliculogenesis and stimulates proliferation and suppresses apoptosis in granulosa cells^35^. We have previously reported that chronic DHT stimulation inhibits GDF9 expression during antral follicle growth^26^. To further examine the follicular stage-dependent androgenic regulation of GDF9 expression and to determine if such responses are related to DHT-induced antral follicle growth arrest *in vivo*, we first compared the relative expression of GDF9 at different stages of follicular development by IF. As shown in Fig. 5A, GDF9 expression in the oocytes of antral (*P* < 0.001) but not of preantral (*P* > 0.05) follicles was markedly down-regulated in DHT-treated rats when compared to control rats. To examine whether antral follicular growth arrest in DHT-treated rats is indeed related androgen-induced GDF9 down-regulation, antral follicles from DHT-treated or control rats were cultured for 4 days in the absence and presence of GDF9 (100 ng/ mL), and the growth was assessed. Antral follicles cultured in the absence of GDF9 exhibited minimal growth (Fig. 5B). However, exogenous GDF9 significantly increased follicle growth in both of group (*P* < 0.01 CTL + GDF9 vs. CTL; *P* < 0.001 DHT + GDF9 vs. DHT Fig. 5B). Our findings suggested that DHT-induced follicle growth arrest may be associated with GDF9 down-regulation and that this phenomenon could be restored by supplementation of GDF9 *in vitro*.

## Discussion

Using a chronically androgenized rat model, we have investigated in the present study the role of RNF6-mediated site-specific AR ubiquitination in androgen-induced antral follicular growth arrest. We have demonstrated that chronic DHT treatment inhibited antral follicle growth during folliculogenesis in the rat *in vivo*. Androgenic stimulation resulted in marked increase in RNF6 content and down-regulation in AR levels in antral follicles. Those two responses closely associated with suppressed AR K63-linked poly-ubiquitination and enhanced AR K48-linked poly-ubiquitination. Additionally, we also have demonstrated that DHT effects were attenuated by gonadotropin treatment *in vivo*. Our studies also support the notion that androgen suppresses antral follicle growth in part by inhibition of sKitlg and GDF9 expression, since antral follicles growth in the DHT-treated rats could be restored by exogenous GDF9 *in vitro*.

PCOS is commonly associated with anovulatory fertility. Features of this syndrome also include hyperandrogenism, insulin resistance, immune disorder and multiple hormone imbalances^7, 36, 37^. It is often manifested with dysregulated folliculogenesis and reduced fecundity^38^. Chronic and abnormally high androgen levels suppress antral follicular development^39^. It has been previously demonstrated that androgen resulted in suppressed antral follicular growth and estrogen secretion in the rats and mice^26, 40, 41^. In addition, testosterone or DHT supplementation in pig oocytes cultures medium significantly increased the proportion of oocytes arrested in the metaphase I stage^42^. Suppressed ovarian follicular maturation has been reported after neonatal androgen administration^43^, as also evident in PCOS^39^. Our current studies clearly show that DHT significantly induces follicular growth arrest in the antral follicle stage but not in the preantral follicles *in vivo*. It suggests that the role of androgen in the regulation of ovarian folliculogenesis is follicle stage-dependent. Specifically, androgen is a negative effector during the antral follicle development.

Although biochemical and mass spectrometry analyses suggest direct interactions between the E3 ligase RNF6 and AR^16^, there is no report on whether RNF6 modulates proteasome degradation of AR or enhances AR transcriptional activity in an androgen-induced PCOS rat model. Our current studies show that DHT increases K48-linked poly-ubiquitination of AR and decreases K63-linked poly-ubiquitination of AR in antral follicles *in vivo*. It suggests that RNF6 plays an important role in androgen-induced AR degradation during antral follicle growth *in vivo*.

GDF9 is important promoter of preantral follicular growth^19–21^ and that its expression is regulated by androgen^24, 31^. In a rat PCOS model, chronic DHT treatment inhibits GDF9 expression and antral follicle growth^26^. Although mRNA abundance of GDF9, but not BMP15, in human oocytes has been reported to be decreased in PCOS^44^, whether antral follicle arrest in PCOS is due to androgen-induced, RNF6-mediated AR ubiquitination and AR down-regulation and subsequent dysregulated GDF9 expression is not known. We have previously demonstrated that androgen up-regulates RNF6, AR and granulosa cell proliferation in pre-antral follicles *in vitro* ^18^ and that RNF6 knockdown or androgen receptor antagonist treatment inhibits the growth of these follicles^45^. In the present studies, we have demonstrated that DHT increased site-specific AR polyubiquitination, AR degradation and decreased GDF9 expression. Moreover, the androgen-induced antral follicle arrest *in vivo* appears in part due to down-regulation of GDF9 expression, since this response could be attenuated by exogenous GDF9 *in vitro*. Taken together, antral follicle growth arrest observed in PCOS may be due to a loss in androgen responsiveness resulting from down-regulation of AR following K48 polyubiquitination and of androgen-induced GDF9 expression.

We have previously demonstrated that RNF6 is a novel positive mediator in androgen-induced site-specific AR polyubiquitination and AR transcriptional activity for s*Kitlg* expression and granulosa cell proliferation in preantral follicles^18^. Although GDF9 and *Kitlg* are known to mediate androgen action on follicular development, the cellular mechanisms involved in the AR ubiquitination are not completely understood. In the rat, Kitlg is expressed in granulosa cells^46, 47^ and its receptor in the oocyte and theca internal cells^48^. These findings raise the interesting possibility that androgen up-regulates *sKitlg* mRNA expression and protein content, and therefore indirectly promotes preantral follicular growth through the expression and action of oocyte-derived GDF9^21, 49^. The dysregulation of these regulatory mechanisms may be important in the ovarian pathology of PCOS. Our observation in the present study that DHT suppresses the expression of sKitlg and GDF9, and antral follicle growth *in vivo* is consistent with this notion. In contrast, Tuck *et al*., reported that granulosa cells of human subjects with PCOS had a greater intensely of KIT-L than normal subjects. These discrepancies may be associated with differences of human PCOS compared to animal models which need to be further assessed^50^.

Gonadotropins stimulate the growth and recruitment of ovarian follicles in the ovary. In early antral follicles, FSH is a cell survival factor that rescues the small antral follicles from apoptosis^39^. It is used commonly in infertility therapy including controlled ovarian stimulation in *in vitro* fertilization and for PCOS patients^22, 45^. It offers a safe and successful treatment option in patients with PCOS with an acceptable risk for multiple gestations^51^. Our previous and present studies show that gonadotropin enhances *sKitlg* mRNA and protein contents on the antral follicles and attenuates DHT-induced antral follicles arrest *in vivo* ^33, 34^. Therefore, defining the action and interaction of gonadotropin and GDF9 could provide important clue for treatment of dysregulated follicular development in PCOS.

**Figure 6:**
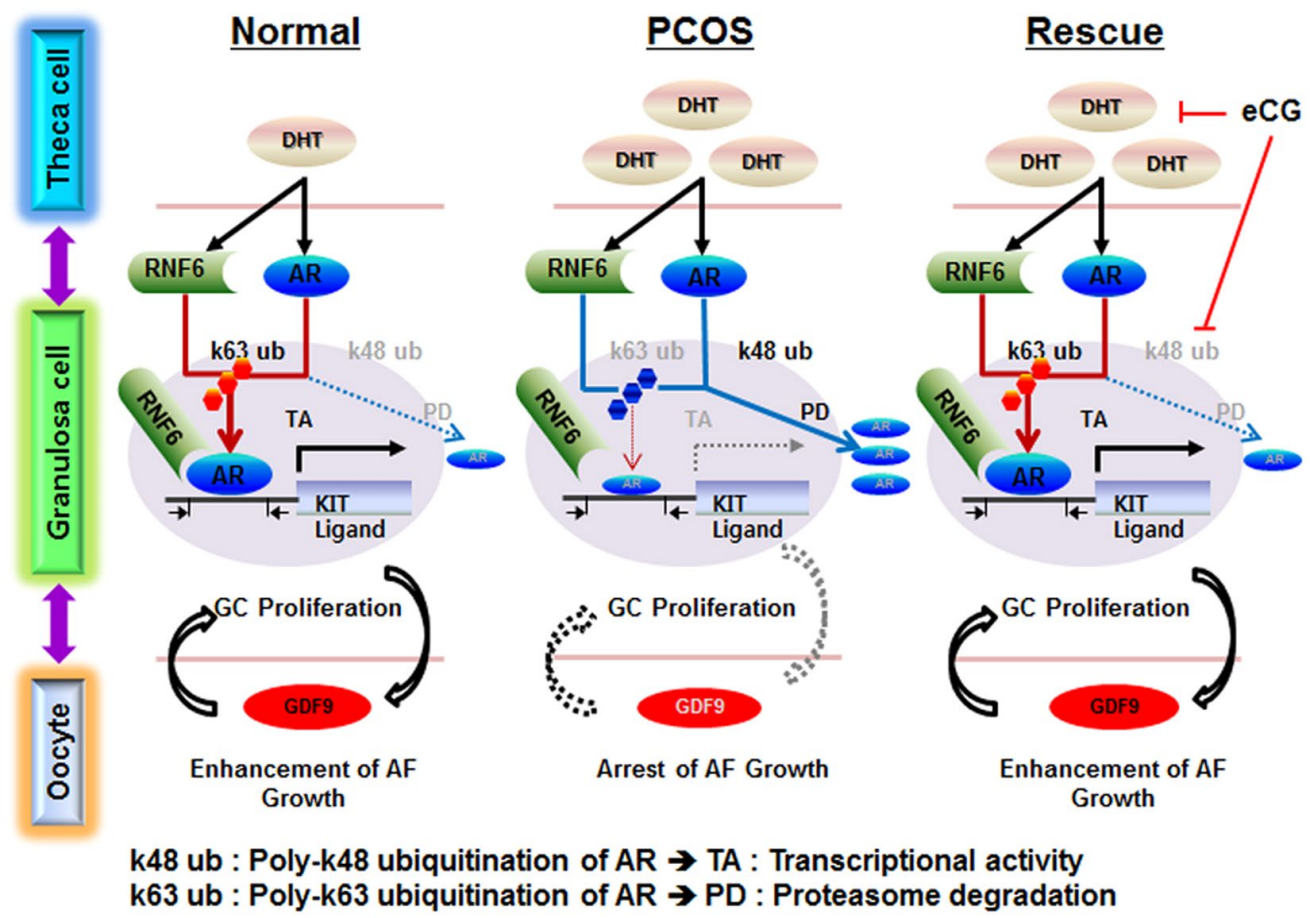
A hypothetical model illustrating RNF6-mediated AR signaling in the control of folliculogenesis and its dysregulation in a PCOS rat model. Androgen increases RNF6-mediated K48 site-specific AR poly-ubiquitination and AR proteasome degradation and decreases AR content in the PCOS rat model. These result in suppressed sKit-L and GDF9 expression, leading to antral follicle growth arrest. Gonadotropin stimulation restores antral follicular growth *in vivo*.

We have previously shown that androgen regulates ovarian follicular growth *in vitro* in a follicle stage-dependent manner by inducing RNF6-mediated, site-specific AR polyubiquitination and altered AR transcriptional activity and stability^45^. Using rat model for PCOS, we have demonstrated that androgen induces antral follicle growth arrest through AR polyubiquitination and down-regulation. To facilitate future investigation on the role of RNF6 in the regulation of folliculogenesis and its deregulation in PCOS, the following hypothetical model is proposed (Fig. 6). Chronic androgen down-regulates AR contents in large antral follicles *via* increased RNF6-mediated K48 site-specific AR poly-ubiquitination and increased AR proteasome degradation. Dysregulation of this pathway suppresses sKITLG and GDF9 expression, leading to growth arrest of the antral follicle. Gonadotropin stimulation *in vivo* restores follicular growth. Although these findings are intriguing, their pathologic significance in human PCOS remains to be explored. Our findings significantly contribute to the current understanding of the role of RNF6 in AR signaling in the control of ovarian follicular growth and its possible dysregulation in PCOS. It provides novel insights in the pathophysiology of PCOS associated patients with androgen excess.

## Electronic supplementary material

Supplementary Information
