# Supplementary material for: Regulation of androgen receptor signaling by ubiquitination during folliculogenesis and its possible dysregulation in polycystic ovarian syndrome
Source: Sci Rep. 2017 Aug 31;7:10272. doi: 10.1038/s41598-017-09880-0 (PMC5578986; doi:10.1038/s41598-017-09880-0)

Supplementary information

Regulation of androgen receptor signaling by ubiquitination during folliculogenesis and its possible dysregulation in polycystic ovarian syndrome

Jung Jin Lim[1,5], Patricia D.A. Lima[1], Reza Salehi [1], Dong Ryul Lee[3,4], Benjamin K. Tsang[1,2]

[1]Department of Obstetrics and Gynecology and Cellular and Molecular Medicine, University of Ottawa, and Chronic Disease Program, Ottawa Hospital Research Institute, Ottawa, Ontario, Canada K1H 8L6

[2]State Key Laboratory of Quality Research in Chinese Medicine, Macau Institute for Applied Research in Medicine and Health, Macau University of Science and Technology, Avenida Wai Long, Taipa, Macao, China;

[3]Fertility Center of CHA Gangnam Medical Center, College of Medicine, CHA University, Seoul, Korea, 135-913

[4]Department of Biomedical Science, College of Life Science, CHA University, Seoul, Korea, 135-081

[5]Department of Biomedical Science, Graduate School of Biomedical Science and Engineering, Hanyang University, Seoul, Korea, 133-791

Supplementary Table 1 List of Antibodies used in current studies.

Primary antibodies

Name	Isotype	Specie cross-reactivity	Supplier	Cat. No.	Dilution in W.B	Dilution in IF
Anti-human RNF6	Rabbit IgG	R, M, H	Abcam	ab-80427	1:1000	1:200
Anti-human AR	Rabbit IgG	R, M	Origene	TA307638	1:1000	1:200
Anti-human Ubiquitin K48	Rabbit IgG	R, M, H	Abcam	ab-190061	1:1000	NA
Anti-mouse Ubiquitin K63	Mouse IgG	R, M, H	Biolegend	#-932202	1:1000	NA
Anti-human GAPDH	Rabbit IgG	R, M, H	Santa cruz	sc-25778	1:8000	NA
Anti-human Kit ligand	Mouse IgG	R, M, H	Santa cruz	sc-13126	NA	1:200
Anti-human GDF9	Goat IgG	R, M, H	Santa cruz	Sc-12244	NA	1:200

Secondary antibodies

Name	Isotype	Dye	Supplier	Cat. No.	W.B	IF
Mouse IgG (H+L)	Goat IgG	Alexa 488	Thermo Fisher	A-11001	NA	1:200
Mouse IgG (H+L)	Goat IgG	Alexa 594	Thermo Fisher	A-11005	NA	1:200
Rabbit IgG (H+L)	Goat IgG	Alexa 680	Life Technologies	A-21077	1:10000	NA
Mouse IgG (H+L)	Goat IgG	DyLight 800	Thermo Fisher	SA5-10176	1:10000	NA

Supplementary Table 2 List of Primers used in the current studies

Gene	Gene bank Accession No.	Primer Sequence (5'-3')	Amplicon size (bp)	Annealing Temp. (°C)
sKIT-L	NM_021843.4	F: TGACCTCGTGGCATGTATGG<br>R: GCTGCAACAGGGGGTAACAT	206	52
GAPDH	XM_017592435.1	F: TGTGAACGGATTTGGCCGTA<br>R: GATGGTGATGGGTTTCCCGT	208	56

Supplementary figure 1 Blot in the Figure 2C and 2D (Do not contract change)

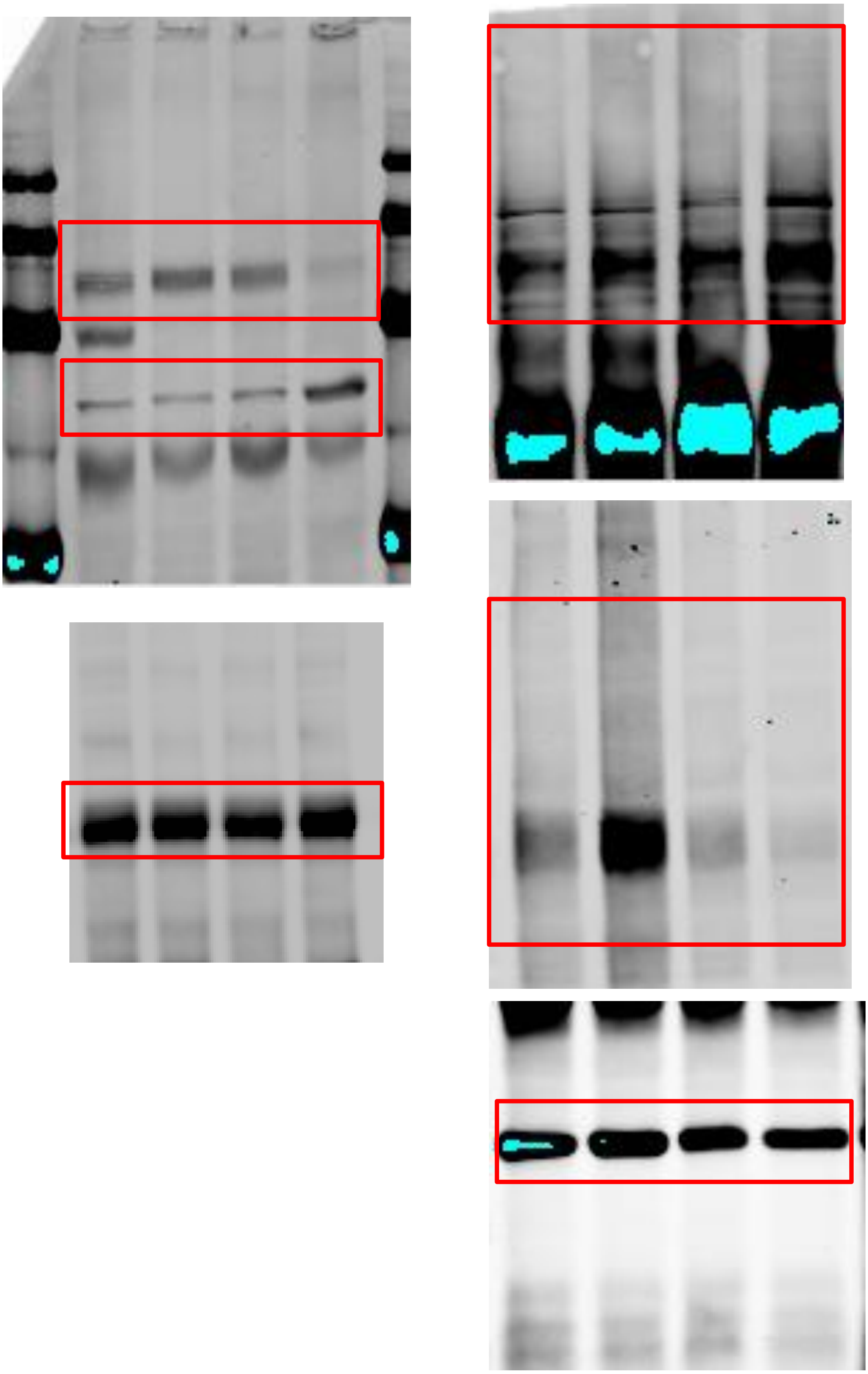

Supplementary figure 1 Blot in the Figure 3A and 3B (Do not contract change)

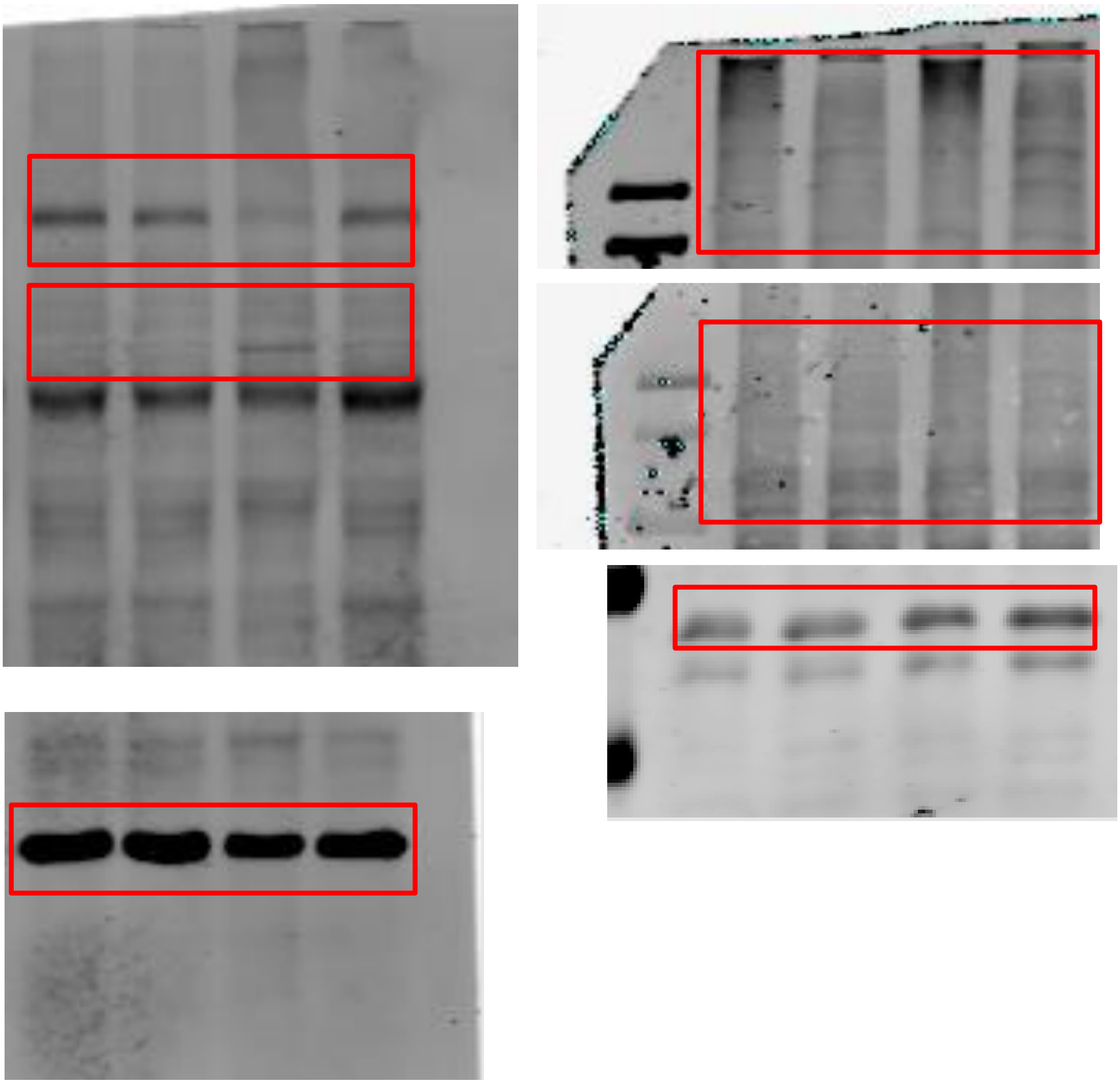

Supplement: Supplementary file 1 — Supplementary Information [file 41598_2017_9880_MOESM1_ESM.pdf]
